# Christian Science

**Published:** 1889-12

**Authors:** 


### CHRISTIAN SCIENCE.

This term, according to the dictionary, means knowledge pertaining to Christ and his religion; therefore, when it is applied to a system of healing the sick, it is misapplied, and we believe that those who so apply it do it with the purpose of misleading. A recent writer, a clergyman of high standing, answers the question—What is Christian Science? thus: "No matter: All mind: All God: All good: No evil. These tenets clearly deny a personal God and nearly every doctrine which Christians hold, including such vital doctrines as redemption, regeneration, sin, resurrection, heaven, and hell, and a personal devil, and affirm absolute inherent perfection of man." This shows conclusively that the title under which those who pretend to believe in this fraud masquerade and who have unluckily succeeded in finding many dupes, is a delusion and snare. The people who teach these false doctrines may be morally good, kind and intelligent in some cases, but they are, as a rule, vain sentimentalists, either ignorant of, or disbelievers in, the Bible, the mainstay of the Christianity they claim to believe. The question naturally arises how we can explain "the many marvelous cures accomplished by Christian science?" They claim to cure all sorts of incurable diseases, as pulmonary consumption, cancer, Bright's disease, leprosy, etc. As a matter of fact they do not cure these diseases. We deny most of their claims. The majority of the ailing do not themselves know what diseases they have; and even skilled physicians are not always positive in their diagnosis. Many of those whose cases are widely trumpeted as cures die afterwards of the disease of which they believe they have been cured, while a great many who are not ill at all, believe themselves to be so, and they, of course, can readily be convinced by these so-called Christian scientists that they are cured. In many diseases, the influence of mind and will over the body, when rightly directed, is of great value in medical treatment, as has been seen in cases treated by mesmerists. There could be no objection to the treatment employed by those who call themselves Christian scientists, provided they would call in regular physicians to effect the curing.—*American Analyst.*

